# Investigating the role of landscape composition on honey bee colony winter mortality: A long-term analysis

**DOI:** 10.1038/s41598-018-30891-y

**Published:** 2018-08-16

**Authors:** Sabrina Kuchling, Ian Kopacka, Elfriede Kalcher-Sommersguter, Michael Schwarz, Karl Crailsheim, Robert Brodschneider

**Affiliations:** 1 Austrian Agency for Health and Food Safety (AGES) GmbH, Data, Statistics and Integrative Risk Assessment, Graz, 8010 Austria; 2 University of Graz, Institute of Biology, Graz, 8010 Austria; 3 Austrian Agency for Health and Food Safety (AGES) GmbH, Data, Statistics and Integrative Risk Assessment, Vienna, 1210 Austria

## Abstract

The health of honey bee colonies is, amongst others, affected by the amount, quality and diversity of available melliferous plants. Since landscape is highly diverse throughout Austria regarding the availability of nutritional resources, we used data from annual surveys on honey bee colony losses ranging over six years to analyse a possible relationship with land use. The data set comprises reports from a total of 6,655 beekeepers and 129,428 wintered honey bee colonies. Regions surrounding the beekeeping operations were assigned to one of six clusters according to their composition of land use categories by use of a hierarchical cluster analysis, allowing a rough distinction between urban regions, regions predominated by semi-natural areas and pastures, and mainly agricultural environments. We ran a Generalised Linear Mixed Model and found winter colony mortality significantly affected by operation size, year, and cluster membership, but also by the interaction of year and cluster membership. Honey bee colonies in regions composed predominantly of semi-natural areas, coniferous forests and pastures had the lowest loss probability in four out of six years, and loss probabilities within these regions were significantly lower in five out of six years compared to those within regions composed predominantly of artificial surfaces, broad-leaved and coniferous forest.

## Introduction

Honey bees (*Apis mellifera*) play an essential role in the ecosystem by pollinating wild and cultivated plants. In recent years, high mortality of honey bee colonies has been reported from many regions of the world^1–5^. The drivers of such losses include several biotic and abiotic factors, and synergistic actions of multiple actors are likewise believed to affect colony health^6–9^.

For wild bees, which greatly depend on their nesting habitats, landscape composition strongly influences their total abundance and species richness, reflected for instance in Simpson’s diversity^10,11^. The nature of the resource providing unit, which is defined as “environmental components around the hive including contaminants”^12^, also affects managed honey bee colonies. Some types of landscape may be better suited for honey bees than others^13^. Against this backdrop, the effect of the honey bees’ exposure to forage plants with agriculturally administered insecticides is of particular interest. The use of neonicotinoid insecticides threatens bees; research on this topic has, however, been surrounded by controversy^14,15^. Ingestion of sublethal doses results in deficiencies in the bees’ performance, which was demonstrated for various parameters including colony development and survival^16–18^. There are only few field studies that focused directly on the effects of insecticide-treated crops on honey bees with at least partially contradictory results^17,19–26^.

Alburaki *et al*. conducted two studies^27,28^ in Quebec, Canada in order to analyse the impact of neonicotinoid pesticides on honey bee health by placing honey bee colonies in different agricultural environments (neonicotinoid-treated and untreated cornfields). The results indicate a higher load of *Varroa destructor* mites and a higher prevalence of black queen cell virus (BQCV) for colonies placed in treated areas, whereas no clear effect was found on weight and brood. Two other studies from Alburaki *et al*.^29,30^ performed in Tennessee, USA also aimed at investigating the effect of landscape and pesticides on honey bee health. While foragers’ mortality did not differ between agricultural and non-agricultural landscapes^30^, differences became apparent in biological traits of honey bee colonies according to the intensity of agricultural use^29^. It should, however, be noted that the number of observed colonies was relatively small (four to thirty-two) and that the maximum period of observation was two years.

Besides exposure to insecticides, land cover influences the wellbeing of honey bee colonies also via the amount, quality and seasonal pattern of nutritional resources provided^31^. Diversity and quality of collected pollen forage affects honey bee health and varies depending on season and landscape composition^32–34^. Danish hive scale data also suggests that colonies gain more honey in landscapes composed of more than 50% urban areas compared to other landscapes^35^.

In many countries, standardised large scale data collection on honey bee colony losses was put into practice only recently^36–38^. The data are based on self-reports by beekeepers and allow epidemiologic analyses of operational drivers such as hive management techniques. Colony loss data can for instance be used to identify best practice of hive management and treatment of colonies against the parasitic mite *Varroa destructor*^2,3,39^. Combinations of such survey based data with other available data sets or further sample analysis have been shown to facilitate our understanding of honey bee health^17,40^.

In Austria, data on winter mortality of honey bee colonies are available from the winter 2007/08 onwards, with increasing sample size and spatial accuracy over the years^36,41^. The number of responses increased over the years and now covers approximately five percent of all registered Austrian beekeepers from each region of the country^38^. A previous study used parts of this data set to investigate how long-term weather conditions influence honey bee colony winter mortality rates. Statistical correlations between monthly climate variables and winter mortality rates demonstrated that warmer and drier weather conditions in the year preceding the winter are generally accompanied by increased loss rates of honey bee colonies during the respective winter^42^.

The only study so far that connected larger data sets of honey bee colony winter losses and land use analysed data from 166 to 188 apiaries for three consecutive winters in Luxembourg^13^. They found 60 out of 133 land cover classes to be correlated with bee colony losses, with the majority of these classes being associated with human activities other than agriculture. The study further suggests that significant effects may be found in some years or certain parts of the country only. In the present study, we investigate the impact of land cover on colony winter mortality in Austria with a much larger dataset for six consecutive years, including two winters with high losses and one winter with a very low loss rate.

## Material and Methods

### Data

The study at hand is based on data from the Austrian monitoring scheme of honey bee winter losses. Within the scope of the COLOSS questionnaire used to collect data, apiarists are annually encouraged to report their winter losses voluntarily and optionally anonymously^37,41^. The gathered information comprises specifications on the location of the beekeeping operation, the total number of wintered colonies and the number of lost colonies (i.e. the sum of dead colonies and colonies with queen problems) for the winters from 2010/11 to 2015/16. The locations of the beekeeping operations were recorded on the level of municipality in order to ensure beekeeper anonymity, i.e. exact coordinates were not available for the analysis. In Austria, municipalities are very heterogeneous in size. The area of the 2,354 municipalities ranges from 11 to 46,680 ha with an average size of 3,563 ha. To facilitate comparability, municipalities were aggregated into more homogeneous regions, resulting in 263 regions with an average size of 31,890 ha. These regions are based on the locations of the meteorological stations, listed in the yearbook of Austria’s Central Institute for Meteorology and Geodynamics (ZAMG^43^), which are evenly distributed across Austria. For the determination of the regions, each municipality was assigned to the nearest meteorological station (based on the distance between municipality centroid and meteorological station) and municipalities assigned to the same station were combined into one region. For each region, the elevation above sea level was determined at its centroid in order to analyse the possible relation between altitude and winter losses.

The questions in the COLOSS questionnaire were slightly adapted over the years. As of 2011, beekeepers were also asked to specify whether they kept migratory colonies, but no further details on the time of migration or the other locations were available. In a pre-analysis, we modelled the winter losses for each year separately and tested the significance of the migratory effect. As the migratory effect was not significant in the majority of the models, we treat all apiaries as if they were stationary. Due to the vague information that is available on migration, no indication is given that our assumption may cause systematic bias in the reported analysis.

**Figure 1:**
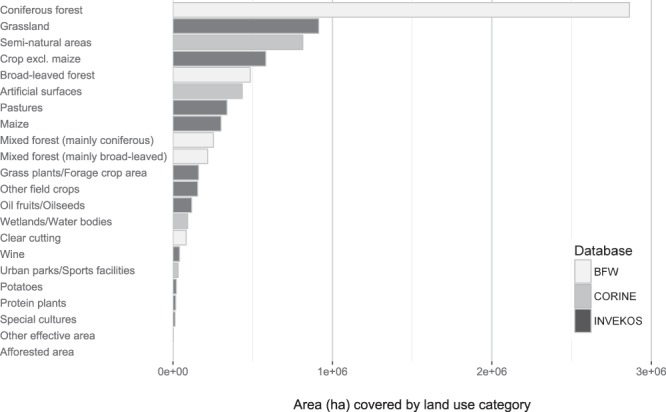
Area (in ha) that can be allotted to land cover categories for 2014. Data from BFW and CORINE categories are constant over the years; the INVEKOS categories may differ slightly in other years according to annual differences in cultivation.

Information on land cover in Austria comprises data from three different data sources: INVEKOS (Integrated management and monitoring system from the Austrian Federal Ministry of Agriculture, Forestry, Environment and Water Management^44^), BFW (Woodland map of the Austrian Research Centre for Forests^45^) and CORINE (Coordination of Information on the Environment CORINE Landcover^46^). INVEKOS provides the most detailed information on agricultural land use in Austria. Approximately 300 land cover categories (e.g. pastures, crop, oil fruits) which are assigned to seven main categories are surveyed and available on a yearly basis. We predominantly used the main categories as defined by INVEKOS. Only land cover subcategories discussed as possibly influencing honey bee health, such as maize^19,26–28^, oil fruits/oilseed^25^, potatoes and protein plants, were kept as separate subcategories. Cereal crops other than maize are contained in a separate category ‘crop excl. maize’. Field crops that do not fall into the categories cereal crops, oil fruits/oilseed, potatoes and protein plants are summarised in a broader category ‘other field crops’. The BFW data set is based on satellite images from the years 2000–2003 and includes the categories ‘coniferous forest’, ‘broad-leaved forest’, ‘mixed forest (mainly coniferous)’, ‘mixed forest (mainly broad-leaved)’ and ‘clearcutting’. The European land cover programme CORINE provides 47 land cover categories on cropland, artificial surfaces, urban facilities (including parks/sports facilities) and wetlands. CORINE data is updated every six years. At the time of the analysis, the most recent data set was based on the reference year 2012. As some categories overlap between the different data sources, we considered cropland categories only from the INVEKOS data, woodlands only from the BFW, and wetlands only from the CORINE data, as these seemed the most reliable sources for the respective categories. Examination of the available land cover categories resulted in 247 relevant categories, which were thematically summarised into 22 main categories. These 22 land cover categories comprise 13 agricultural land use categories from the INVEKOS data set, 5 woodland categories from the Austrian Research Centre for Forests (BFW), and 4 further categories from the CORINE data set: wetlands/water bodies, artificial surfaces, urban parks/sport facilities, and semi-natural areas. Semi-natural areas include the sub-categories ‘Scrub and/or herbaceous vegetation associations’, ‘Open spaces with little or no vegetation’ and the unclassified land/water areas. Figure 1 lists the considered land use categories and shows the total area covered by each category in 2014. This figure shows that Austria is to a large extent covered by coniferous forests, followed by grassland and semi-natural habitats.

### Statistical analysis

The relation of land use and honey bee winter losses was modelled using a Generalised Linear Mixed Model (GLMM). The unit of observation was one colony within a beekeeping operation. Hence, information on winter losses could be considered as a binary response (success/failure of overwintering). Consequently, the binomial distribution and the logit-link function were assumed for the model. As the land use categories are highly correlated, see Supplementary Figures S1–S2, they were not directly used as explanatory variables in the model. Instead, a hierarchical cluster analysis was conducted to group regions that show similarities regarding their land cover composition. Similarity was defined by means of correlation-based distance matrices^47^. Two regions are considered as being related if land use in these regions is highly correlated. For the land use categories associated with cropland, data was available on a yearly basis. Therefore each year-region combination was considered for the cluster analysis. The clustering was performed hierarchically, i.e., initially, each region forms its own cluster. Successively, the two most related clusters are combined in each step until only one cluster remains^47^. This process can then be visualised in the form of a dendrogram, which was used to select a complete partitioning of six clusters for further analysis. Each beekeeping operation was then allocated to one of the six clusters according to the land cover composition in its respective region. For each cluster, the proportion of lost colonies and corresponding 95% exact Clopper-Pearson confidence intervals^48^ were determined in a first descriptive analysis. In the subsequent multivariate model, the cluster membership of each operation was considered as a possible risk factor for winter losses.

Although land use was the main variable of interest, winter losses may further be influenced by a complex combination of different risk factors. In order to consider other sources of risk, the total number of wintered colonies, the municipality size and the elevation above sea level of the region in which the apiaries are located were considered as confounder variables. The size of the municipality was evenly categorised into four classes according to the corresponding quartiles and considered as a factor in the model. The classes are as follows:

- very small, if the overall area of municipality ≤1,620 ha
- small, if the overall area of municipality ∈(1,620 ha; 2,800 ha]
- medium, if the overall area of municipality ∈(2,800 ha; 4,700 ha]
- large, if the overall area of municipality >4,700 ha.

Switanek *et al*.^42^ showed the climate to be among the relevant factors for honey bee winter mortality. In particular, the study results indicate that the weather conditions in the preceding year have a significant influence on winter mortality, and warmer and drier weather was associated with higher losses. In our model, however, information on weather conditions was not available for the whole study period and, hence, not incorporated in the model. As weather conditions were not the main interest factor of the study at hand, the year of observation was considered as an additional risk factor to account for different unobserved environmental conditions. In order to deal with possible overdispersion in the data, an ID was assigned to each data set within a year and this ID was considered as a random effect in the model.

Model selection was performed through forward selection; the Akaike Information Criterion (AIC) and likelihood-ratio tests served as model selection criteria. Significant factors were identified at the significance level α = 0.05. The model selection process is further described in Supplementary Table S1. All statistical analyses were performed in R, version 3.3.2^49^ using the R packages binGroup^48^, lme4^50^ and ggplot2^51^.

## Results and Discussion

### Honey bee winter losses

The available data from questionnaires on honey bee winter mortality comprises reports from a total of 6,670 beekeepers. Observations from 15 beekeepers were excluded due to missing information on beekeeping operation location. The remaining beekeepers in total wintered 129,428 colonies between the winters of 2010/11 and 2015/16. The lowest number of reporting beekeepers was 559 in the winter of 2010/11; the winter of 2011/12 had the highest coverage with 1,528 reporting beekeepers (Table 1). As participation was voluntary and the data were recorded anonymously, it was not possible to track specific beekeeping operations across the years. Table 1 shows the number of reporting operations for each winter along with the total number of wintered and lost colonies and the relative proportion of lost colonies including a 95% confidence interval. Furthermore, the table shows that winter losses vary considerably from year to year ranging from 8.10% to 28.41%. The winters of 2011/12 and 2014/15 stand out with very high winter losses (26.01% and 28.41% respectively), whereas winter 2015/16 showed the lowest observed loss rate (8.10%) within the total observation period.

**Table 1:** Number of reporting operations, number of reported wintered and lost honey bee colonies.

Winter Season	Reporting operations	Wintered colonies	Lost colonies	Relative Proportion of lost colonies (in %)	95% Confidence interval for relative proportion
2010/11	559	12,809	2,037	15.90	[15.27;16.55]
2011/12	1,528	32,119	8,354	26.01	[25.53;26.49]
2012/13	997	19,406	3,363	17.33	[16.80;17.87]
2013/14	1,023	18,794	2,404	12.79	[12.32;13.28]
2014/15	1,259	22,882	6,501	28.41	[27.83;29.00]
2015/16	1,289	23,418	1,896	8.10	[7.75; 8.45]
Overall	6,655	129,428	24,555	18.97	[18.76;19.19]

Lee *et al*.^52^ discussed the relevance of operation size and associated management factors (e.g. tendency for migratory beekeeping and the willingness or ability to treat bees for bee pests and diseases). Following Lee *et al*.^52^, beekeepers can be categorized as ‘backyard beekeepers’ (1–50 colonies), ‘sideline beekeepers’ (51–500 colonies) and ‘commercial beekeepers’ (more than 500 colonies) according to the number of colonies they have. They found that winter losses tend to be lower for commercial beekeepers than for backyard beekeepers. Our data contains observations from 6,223 backyard beekeepers, 430 sideline beekeepers and two commercial beekeepers. The average size of the observed beekeeping operations was approximately 19 colonies. Operations with more than 500 colonies are rare in Austria^36^; the largest beekeeping operation in our data set managed 580 colonies. Although Austrian commercial beekeepers generally have smaller colony numbers than their counterparts in the US, operation size is nevertheless assumed to be a risk factor associated with winter losses also in Europe^38^ and our model results (see below) confirm the importance of operation size for winter losses.

### Cluster analysis

**Figure 2:**
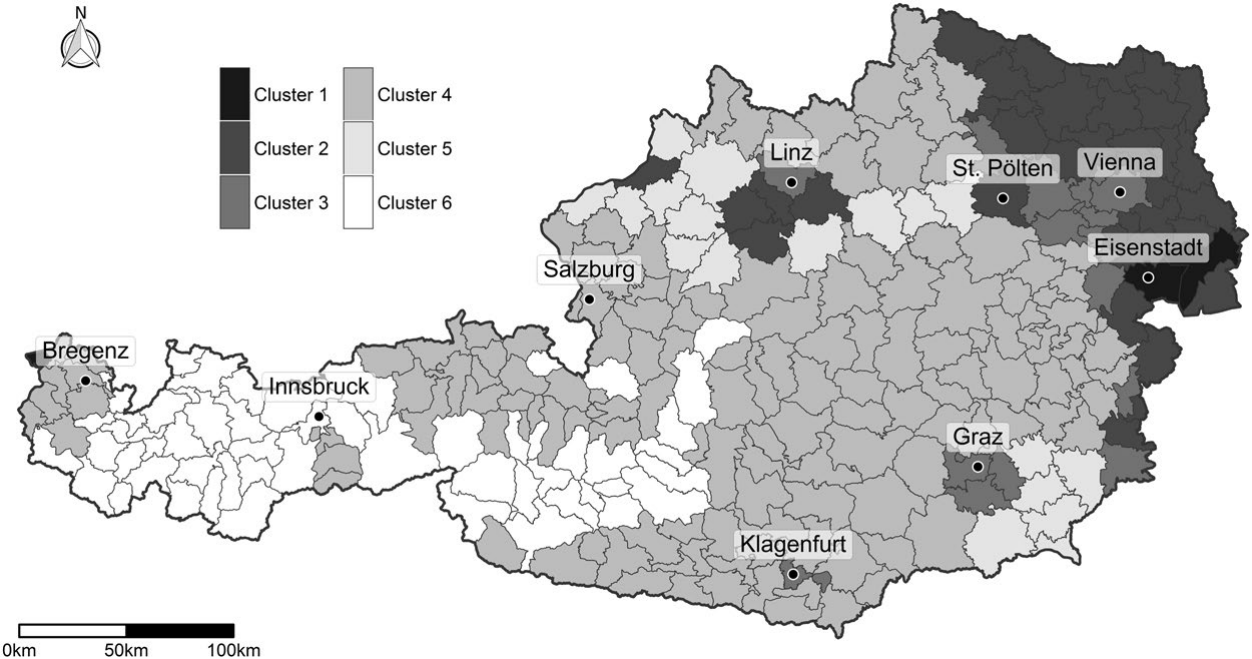
All considered regions in Austria and their cluster membership. Geographical distribution of the six identified clusters in Austria.

For each beekeeping operation, geographic information was available on the level of municipality. In order to guarantee the anonymity of beekeepers in the study at hand, data was collected on operation level^37^, whereas one operation could also have several apiaries. Therefore, it was not possible to exactly evaluate the land cover in the estimated flight range around each honey bee colony. The accuracy of the results depends on the assumption that the assignment of the apiaries to the clusters based on the available geographical information is correct. Furthermore, the observations do not follow a designed sampling plan, as the high number of observed colonies across Austria could only be ensured through a voluntary self-reporting system regarding wintering success. Representativeness can therefore not be guaranteed and the presence of a reporting bias can neither be ruled out nor quantified. Therefore, and also because honey bees forage on an area of up to 100 km^2^ (according to Couvillon & Ratnieks, 2015^53^), the precise landscapes in which honey bees collect food cannot be determined. Furthermore, honey bees do not randomly forage over a landscape but exploit nested patches of forage due to their dance communication. The land cover in Austria is very heterogeneous at a small scale and the individual land use categories are highly correlated, see Supplementary Figures S1–S2. The grouping of individual land use categories into a manageable number of clusters removes the strong correlations that would otherwise impair the stability and interpretability of the statistical analysis. The hierarchical cluster analysis was hence used to assign each of the 263 regions of Austria into one of six clusters according to the proportional composition of the land use categories in the region. Clusters were identified such that regions in the same cluster are more similar regarding their land cover composition compared to regions from different clusters. Table 2 can be used to identify predominant land cover categories for each cluster. It contains the median relative area for each cluster and land cover category (in %). Figure 2 displays the spatial distribution of the six distinct clusters of land use within Austria. As clusters are characterised by the proportional composition of land use categories, these categories themselves cannot be assigned to one cluster exclusively. The cluster analysis rather defines weights, with which the categories are present in each cluster. This is illustrated by the composition of Cluster 2 (Table 2) which contains crop excl. maize (29.63%), broad-leaved forest (9.88%), other field crops (9.54%) and artificial surfaces (7.25%). Cluster 5 predominantly contains grassland (15.72%), coniferous forest (14.85%), maize (13.32%) and crop excl. maize (12.40%). In the remaining document, generated clusters will be referred to by their dominant land cover categories (i.e. categories with median values greater than 10%) to facilitate the identification of the most influential land use categories.

**Table 2:** Median relative area of land cover category per cluster (in %). The land cover categories are sorted by the total covered area in Austria. Categories with median value greater than 10% are highlighted for each cluster.

Land cover category	Cluster 1	Cluster 2	Cluster 3	Cluster 4	Cluster 5	Cluster 6
Coniferous forest	0.91	2.33	**12**.**73**	**46**.**38**	**14**.**85**	**31**.**92**
Grassland	3.18	1.79	6.03	**12**.**90**	**15**.**72**	5.68
Semi-natural areas	0.00	0.00	0.00	2.65	0.00	**33**.**12**
Crop excl. maize	**15**.**38**	**29**.**63**	5.61	0.31	**12**.**40**	0.00
Broad-leaved forest	5.82	9.88	**16**.**10**	3.45	7.18	0.64
Artificial surfaces	8.40	7.25	**18**.**60**	3.61	6.65	1.55
Pastures	0.00	0.00	0.00	2.47	0.00	**11**.**60**
Maize	2.82	6.10	4.04	0.55	**13**.**32**	0.00
Mixed forest (mainly coniferous)	0.21	0.20	3.96	3.63	3.50	0.56
Mixed forest (mainly broad-leaved)	0.47	0.62	4.17	2.88	3.32	0.51
Grass plants/Forage crop area	4.12	1.80	2.18	1.01	3.52	0.03
Other field crops	3.14	9.54	1.91	0.06	1.62	0.00
Oil fruits/Oilseeds	4.49	6.56	1.65	0.00	2.11	0.00
Wetlands/Water bodies	**27**.**34**	0.07	0.31	0.18	1.24	0.25
Clear cutting	0.90	0.68	1.06	0.23	1.27	0.00
Wine	5.04	0.63	0.10	0.00	0.00	0.00
Urban parks/Sports facilities	0.36	0.16	0.36	0.19	0.13	0.32
Potatoes	0.06	0.54	0.02	0.01	0.04	0.00
Protein plants	0.63	0.70	0.22	0.00	0.26	0.00
Special cultures	0.28	0.14	0.07	0.00	0.07	0.00
Other effective area	0.03	0.03	0.01	0.00	0.02	0.00
Afforested area	0.00	0.00	0.00	0.00	0.00	0.00

Cluster 1, which is dominated by wetland areas and crop excl. maize, covers only three of the 263 regions in Austria. Said regions are all located near major Austrian lakes (see Fig. 2): two of the regions are located near Lake Neusiedl (Burgenland), one region lies near Lake Constance (Vorarlberg). The geographic distribution of the three regions further demonstrates that spatial vicinity of regions did not in any way influence the generation of the clusters. Cluster 2 is dominated by crop cultivating areas. The predominant land use categories in Cluster 3 are artificial surfaces, broad-leaved forests and coniferous forests. The geographic distribution of the associated regions shows that these regions are located around major cities (Vienna, Linz, Graz, Klagenfurt), this explains the dominance of artificial surfaces in this cluster. Cluster 4, predominated by coniferous forest and grassland, comprises the largest number of regions. Cluster 5 includes many areas used for cultivating crop and maize as well as larger areas of grassland and coniferous forests. The regions that form Cluster 6 are mainly located in mountainous areas. The dominating land use categories in this cluster are semi-natural areas, coniferous forest and pastures. The cluster, however, does not contain any categories related to agricultural cultivation (median coverage of 0% for such categories, Table 2).

**Figure 3:**
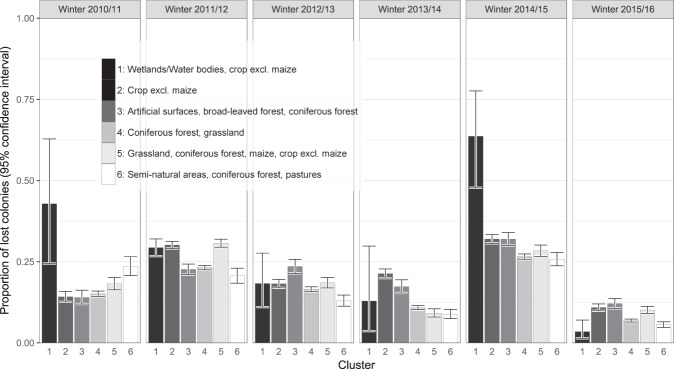
Proportion of lost colonies for each identified cluster and each observed year including corresponding 95% confidence intervals.

The study at hand aims at examining the relationship between the land use composition surrounding the location of bee colonies and honey bee winter losses. This was achieved by analysing the (statistical) influence of the landscape cluster in which colonies are located on the winter losses of the respective operation. Figure 3 provides a first descriptive univariate analysis of the winter loss rates across the clusters. The error bars indicate 95% confidence intervals for the proportion of lost colonies. The width of the error bars is an indicator of the number of observations per cluster/year, as the width of the confidence intervals decreases with increasing observation numbers. Cluster 1 shows exceptionally wide confidence intervals, especially in winters 2010/11, 2013/14 and 2014/15. In these years, the number of reporting operations in this cluster was between 1 and 6 only. The conspicuously high proportion of losses in 2010/11 is thus based on only one report from a single operation with 28 wintered and 12 lost colonies. The losses in 2014/15 are based on observations from six beekeepers, where two of them lost 100% and 86% of their wintered colonies, respectively. The high proportion of winter losses in this cluster is therefore subject to substantial uncertainty due to low sample size. In contrast, Cluster 4 comprises reports from between 313 and 697 operations per year. Based on the descriptive analysis in Fig. 3, no general trend can be identified over the years and no obvious categorisation into clusters with beneficial or detrimental landscape compositions for honey bee colony wintering success can be carried out holding true for all years.

### Relation of land use and colony losses

Using a Generalised Linear Mixed Model, the cluster membership of the beekeeping operations was identified as a significant factor influencing their proportional winter losses. Apart from the cluster membership, operation size, the year of wintering and the interaction of year and cluster membership could be further established as significant factors. The significance of the interaction term indicates that the effect of one cluster may change over the years (e.g. higher winter losses in Cluster 6 compared to Cluster 4 for winter 2010/11, but lower winter losses in Cluster 6 compared to Cluster 4 for winter 2011/12). The model results are listed in Table 3. The estimate for winter losses of an average sized beekeeping operation in Cluster 4 and winter 2010/11 is 12.27%. Compared to Cluster 4, the odds for a winter loss increase by a factor of 2.083 for operations in Cluster 6 for this same winter. Model results further indicate that the risk of a winter loss significantly decreases with the number of wintered colonies. Note that, since the operation size was transformed for the modelling (centered by its mean and scaled by its standard deviation), it is not possible to directly interpret the parameter estimates for a one-unit-increase in operation size. The intercept represents the baseline for an average sized beekeeping operation (approximately 19 colonies). To compute the expected winter loss rates related to other operation sizes, one has to fall back on the transformed variable. The estimated probability for a winter loss for operations with 20 colonies is 12.23% and therefore 0.04% lower than for average sized operations. The probability for a beekeeper with approximately 59 colonies (small sideline beekeeper) is 10.79% and therefore 1.48% lower than for an average sized operation (backyard beekeeper)^38^.

**Table 3:** Estimated model coefficients of the Generalized Linear Mixed Model for the winter losses of honey bee colonies in Austria.

	Estimate	Std. Error	p-value	Odds
Intercept	−1.967	0.103	0.001	0.140
Operation size	−0.109	0.020	0.001	0.897
Cluster membership (reference cluster 4)				
Cluster 1	1.698	1.586	0.284	5.462
Cluster 2	−0.086	0.227	0.703	0.917
Cluster 3	−0.238	0.274	0.385	0.788
Cluster 5	0.418	0.263	0.111	1.519
Cluster 6	0.734	0.274	0.007	2.083
Year of wintering (reference 2010/11)				
2011/12	0.521	0.123	0.001	1.683
2012/13	−0.026	0.132	0.844	0.974
2013/14	−0.638	0.134	0.001	0.528
2014/15	0.756	0.126	0.001	2.130
2015/16	−1.184	0.135	0.001	0.306
Year/cluster interaction (reference 2010/11; Cluster 4)				
2011/12; Cluster 1	−1.079	1.606	0.502	0.340
2011/12; Cluster 2	0.447	0.255	0.080	1.564
2011/12; Cluster 3	0.690	0.320	0.031	1.993
2011/12; Cluster 5	−0.007	0.297	0.982	0.993
2011/12; Cluster 6	−1.183	0.365	0.001	0.306
2012/13; Cluster 1	−1.888	1.875	0.314	0.151
2012/13; Cluster 2	−0.078	0.284	0.783	0.925
2012/13; Cluster 3	0.520	0.340	0.126	1.682
2012/13; Cluster 5	−0.633	0.321	0.049	0.531
2012/13; Cluster 6	−0.841	0.358	0.019	0.431
2013/14; Cluster 1	−1.056	2.279	0.643	0.348
2013/14; Cluster 2	0.827	0.277	0.003	2.286
2013/14; Cluster 3	0.819	0.340	0.016	2.268
2013/14; Cluster 5	−0.456	0.336	0.175	0.634
2013/14; Cluster 6	−1.204	0.368	0.001	0.300
2014/15; Cluster 1	−0.540	1.785	0.762	0.583
2014/15; Cluster 2	0.502	0.267	0.060	1.653
2014/15; Cluster 3	0.889	0.314	0.005	2.433
2014/15; Cluster 5	−0.323	0.309	0.297	0.724
2014/15; Cluster 6	−0.875	0.343	0.011	0.417
2015/16; Cluster 1	−1.715	1.942	0.377	0.180
2015/16; Cluster 2	0.729	0.279	0.009	2.073
2015/16; Cluster 3	0.881	0.333	0.008	2.414
2015/16; Cluster 5	0.125	0.314	0.691	1.133
2015/16; Cluster 6	−0.938	0.336	0.005	0.391

**Figure 4:**
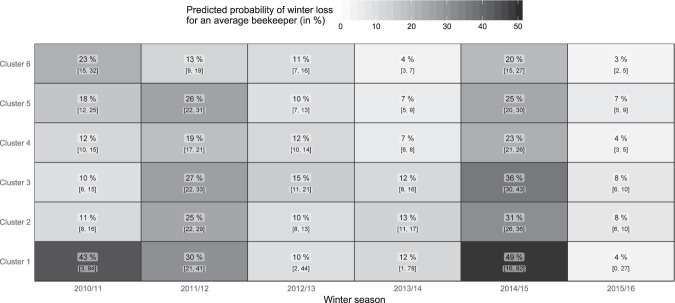
Predicted probability of a winter loss for an average beekeeper with an average number of colonies per cluster and winter season. Probabilities and 95% confidence intervals are stated in each rectangle.

To visualise the interaction effect of year and cluster membership, the predicted probabilities of a winter loss for an average sized beekeeping operation are shown in Fig. 4 in the form of a heat map. Darker colours indicate high probabilities for a winter loss, light colours indicate low probabilities. The values of the estimated probabilities and their 95% confidence intervals are further indicated in the respective tiles. The confidence intervals for the predictions were determined on the link scale using the normal approximation. Uncertainty of random parameters was not taken into account. Cluster 6, with mainly semi-natural areas, coniferous forests and pastures, shows the lowest winter loss probability over nearly all years. Only the winter of 2010/11 contradicts these results with relatively high winter losses in Cluster 6. The semi-natural areas forming Cluster 6 are mainly found in the western, mountainous parts of Austria. In this region we can expect the resource providing unit to show several differences to those in the rest of the country. Differences may relate to the intensity of agricultural use (including pesticide application)^29,30^, the forage diversity^32,35,54^ and climate^42^. It can be assumed that one of the aforementioned factors, or possibly the combination of all, could benefit the bees in semi-natural areas. To finally answer these questions, however, further georeferenced studies are necessary^55^.

Differences between clusters are higher for winters with generally high winter losses (2010/11, 2014/15). This might indicate that the surrounding landscape effects honey bee health especially in years with poor conditions for successful wintering such as high infestation levels with *Varroa destructor*. In years with very low winter losses, on the other hand, the spectrum to detect differences between diverging areas is narrow. The highest risk of winter losses is predicted for Cluster 1 in the years 2010/11 and 2014/15. The estimates for this cluster, which is dominated by wetland areas, are, however, based on very few observations from a low number of beekeepers. The descriptive analysis already showed high uncertainty. This is again reflected in the width of the confidence intervals in Fig. 4, especially for seasons 2010/11 and 2014/15. Results for this cluster must, therefore, be interpreted with caution. Cluster 3, which is characterized by artificial surfaces and forests, shows rather high probabilities for winter losses in most of the years. As was noted for Cluster 6, the results for season 2010/11 also contradict the results of the other reported winter seasons in Cluster 3. The number of observations that these results are based upon is, however, noticeably lower in this year compared to other years. This may challenge the reliability of the results, indicating a strong effect of individual years.

Contrary to what might have been expected from other studies^17,19,20,25,29,30^, Clusters 2 and 5, which comprise regions with relatively high proportions of arable land (Table 2), were not among the clusters with the highest risks of winter losses in most of the years (Fig. 4). Though most of these studies primarily focused on different pesticide treatments of agricultural crops, the general influence of agriculture on honey bee health is poorly studied^12^. It should be noted that in Clusters 2 and 5, arable land makes up less than half of the land cover. The heterogeneity of these clusters and the lack of information on whether the arable land was cultivated organically or conventionally did not allow an assessment of the use of pesticides, which is discussed as a risk factor for honey bee colony winter losses^21^. One explanation for our deviating findings could be the fact that agriculture in Austria is at a small scale compared to that of many other countries. This is also underlined by the heterogeneous composition of Clusters 2 and 5 (Table 2). The influence of such heterogeneous clusters on honey bee health is more difficult to interpret than that of more homogeneously composed clusters such as Cluster 6, which in total consists of more than 70% semi-natural areas, coniferous forest and pastures. Field studies, on the other hand, often face the problem of a limited sample size and the proof that control and exposure group experienced different treatments^19,20,25^. Likewise, our datasets lack information on pesticide usage not only for the INVEKOS dataset, so that no differentiation between the presumably dominant conventional and the less frequent organic farming is possible, but also for all other land use categories that could be contaminated to different extents. Honey bees can forage in large areas, and preferably visit attractive foraging areas^53^. The role of melliferous agricultural crops for honey bee health therefore deserves further research attention, as it applies to beneficial^56^ (plentiful resources during flowering) but also detrimental properties, such as reduced plant diversity^57^ and pesticide exposure, even by drift to neighbouring plants^55^.

As indicated by the significance of the interaction between year and cluster membership, winter losses in one cluster can be higher in one year and lower in another compared to other clusters. Similar effects were shown in Clermont *et al*.^13^, where cluster analysis was used to categorise the 2 km and 5 km radius, respectively, around honey bee apiaries according to land cover. Univariate tests for different years in their study revealed that, e.g., mixed woodland can have a positive impact on successful wintering in one year and a negative impact in other years. A controlled study reported in Alaux *et al*.^54^, comparing agricultural habitats to such enriched with melliferous catch crops, indicates that honey bee health, and thus successful wintering, hinges on the quantity and quality of food supply before wintering. According to another study that placed colonies in landscape dominated by agricultural cultivation, the amount and diversity of pollen available to honey bee colonies was found to be rather independent of landscape composition^58^. However, there is a general agreement that pollen supply is strongly influenced by seasonal variations^32,33^. Apart from the habitat surrounding the colony and forage availability, other factors are assumed to influence winter losses as well^2–6^. These are often related to hive management, but also to environmental factors such as weather^42^, colony density as a driver for pathogen spread^59^ or pesticides^25,55^. This complex interplay of influencing factors and landscape composition, including infestation level with the mite *Varroa destructor*^60^, could explain the strong effect of the year and the varying cluster effects over the years seen in our model results. As there might be different mechanisms, or combinations of mechanisms, effective in a year with high losses compared to a year with low losses, it might be promising to investigate more cases of high winter losses or extend the methodology applied to other countries.

As discussed in Clermont *et al*.^13^, the reference year of the collected land cover data is crucial for the reliability of the analysis. The data basis at hand is composed of three different data sources for land cover^44–46^. The BFW data set, which comprises information on forests in Austria, relies on observations from 2000–2003. Thus, observations regarding woodlands in Austria of 2000–2003 are used to explain winter losses from 2010 to 2015. The CORINE data set, which is mainly used to identify categories such as wetlands and artificial surfaces, was collated in 2012. Both, the BFW and CORINE data, are however assumed to be rather stable over the study period. The INVEKOS data, on the other hand, which characterises the agricultural land cover, is assumed to be volatile compared to the other data sources. This data was available on a yearly basis. Thus, the agricultural land cover in the spring of a year was indeed available to be used to explain the honey bee winter losses in the following winter. The large annual variability in winter loss rates of honey bee colonies experienced during the investigated years might therefore be rather caused by weather and other factors. Our study is a correlational analysis, thus preventing a clear causal explanation. Nevertheless, it facilitates discussions on which habitats are beneficial or disadvantageous for healthy honey bee colonies. In contrast to field studies on locations picked for testing the impact of agricultural versus non-agricultural landscapes or pesticide effects on honey bees, our epidemiological approach allows to investigate the impact of the mixed environments bees are confronted with in a heterogeneous landscape.

## Conclusions

We could show that land cover did affect the honey bee winter colony mortality in Austria, as the estimated loss probability was lowest in regions composed mainly of semi-natural areas, coniferous forests and pastures in four out of six years compared to other regions. Moreover, we found significantly lower loss probabilities in these regions compared to regions predominantly composed of artificial surfaces, broad-leaved forest and coniferous forest in five out of six years. In addition to the land cover, the year of observation, the interaction between the year of wintering and cluster membership, and operation size had significant effects on the winter mortality. This suggests that other factors in addition to landscape composition might be responsible for high winter losses in individual years. It also indicates that conclusions drawn from analyses of a single winter should be interpreted with great caution, and further long-term studies are needed to understand honey bee colony losses.

## Electronic supplementary material

Supplementary Information

## Data Availability

The data regarding the winter losses are not publicly available to preserve the privacy of participants; however they are available from the authors upon reasonable request. The INVEKOS and BFW data are available from the Austrian Federal Ministry of Agriculture, Forestry, Environment and Water Management and the Austrian Research Centre for Forests, respectively, but restrictions apply to the availability of these data, which were used under license for the current study, and so are not publicly available. Data are however available from the authors upon reasonable request and with permission of the aforementioned third parties. The CORINE land cover data are publicly available from the European Environment Agency.
