# Supplementary material for: Investigating the role of landscape composition on honey bee colony winter mortality: A long-term analysis
Source: Sci Rep. 2018 Aug 16;8:12263. doi: 10.1038/s41598-018-30891-y (PMC6095838; doi:10.1038/s41598-018-30891-y)
Supplement: Supplementary file 1 — Supplementary Information [file 41598_2018_30891_MOESM1_ESM.pdf]

Supplementary Information

Investigating the role of landscape composition on honey bee colony winter mortality: A long-term analysis

Sabrina Kuchling[1], Ian Kopacka[1], Elfriede Kalcher-Sommersguter[2], Michael Schwarz[3], Karl Crailsheim[2] and Robert Brodschneider[2]

[1]Austrian Agency for Health and Food Safety (AGES) GmbH, Data, Statistics and Integrative Risk Assessment, Graz, 8010, Austria
[2]University of Graz, Institute of Biology, Graz, 8010, Austria
[3]Austrian Agency for Health and Food Safety (AGES) GmbH, Data, Statistics and Integrative Risk Assessment, Vienna, 1210, Austria

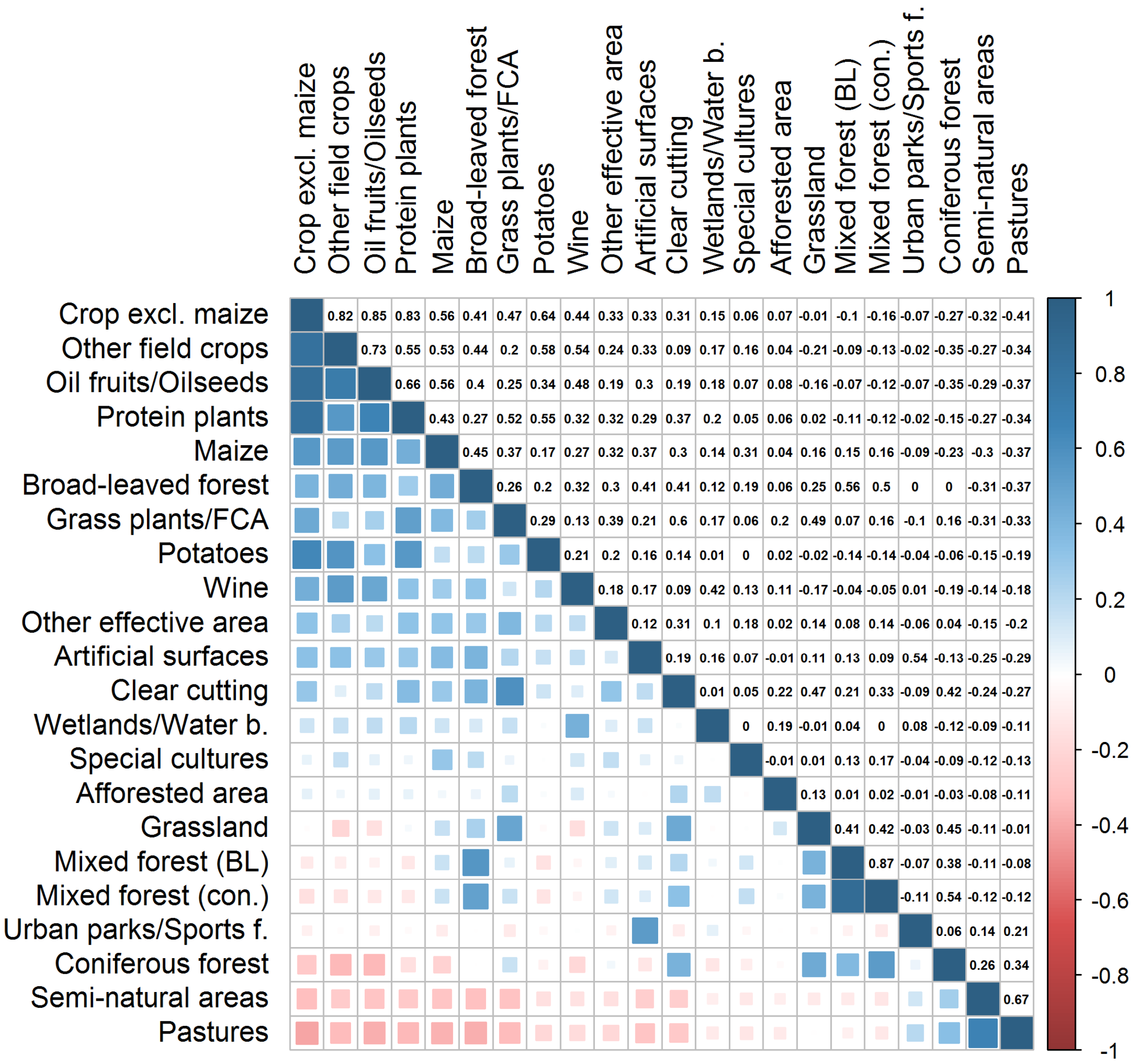

Supplementary Figure S1: Correlation matrix of the land use categories in Austria, exemplary for the year 2014

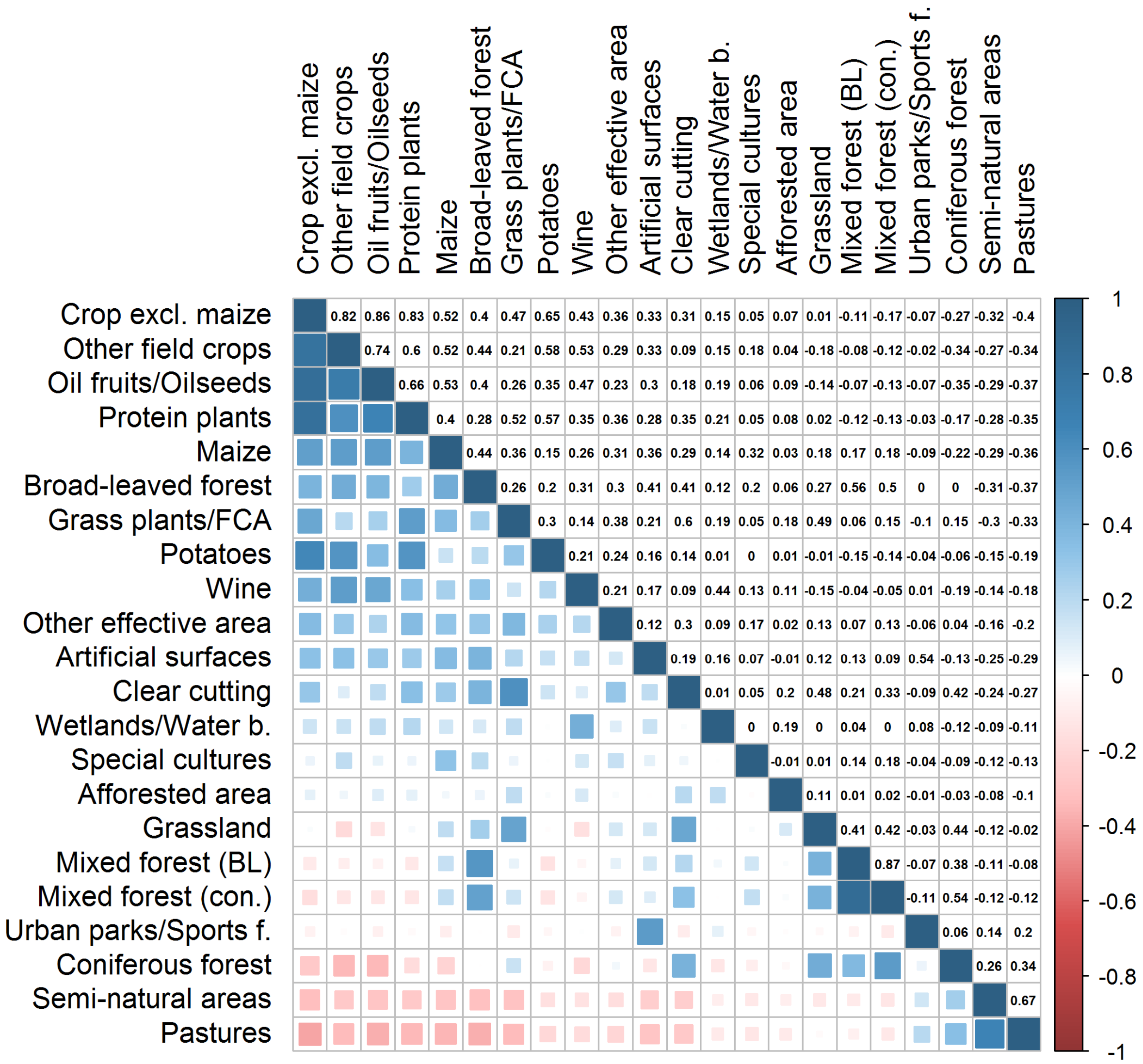

Supplementary Figure S2: Correlation matrix of the land use categories in Austria, aggregated data from 2010–2015

Supplementary Table S1: Akaike Information Criteria for the model selection process; the selected models are highlighted in bold print for each selection step.

Model	p-Value LR-Test	AIC	Change in AIC	Df
Step 0				
Intercept only		28337.21		2
Step 1				
Intercept + operation size	<0.001	28314.54	22.67	3
Intercept + cluster membership	<0.001	28241.06	96.15	7
Intercept + elevation above sea level	<0.001	28212.85	124.36	3
Intercept + municipality size	0.030	28334.29	2.92	5
Intercept + year of wintering	<0.001	27542.18	795.03	7
**Intercept + interaction (cluster membership, year of wintering)**	<0.001	**27457.29**	**879.92**	37
Step 2				
**Intercept + interaction (cluster membership, year of wintering) + operation size**	<0.001	**27430.76**	**26.53**	38
Intercept + interaction (cluster membership, year of wintering) + elevation above sea level	<0.001	27436.20	21.09	38
Intercept + interaction (cluster membership, year of wintering) + municipality size	0.858	27462.53	-5.24	40
Step 3				
Intercept + interaction (cluster membership, year of wintering) + operation size + elevation above sea level	convergence issues due to correlation of cluster membership and elevation above sea level			
